# The Role of Non-Coding RNAs in Breast Cancer Drug Resistance

**DOI:** 10.3389/fonc.2021.702082

**Published:** 2021-09-13

**Authors:** Jin-hai Tian, Shi-hai Liu, Chuan-yang Yu, Li-gang Wu, Li-bin Wang

**Affiliations:** ^1^The Biochip Research Center, General Hospital of Ningxia Medical University, Yinchuan, China, Yinchuan, China; ^2^The Clinical Medicine College of Ningxia Medical University, Yinchuan, China; ^3^Medical Research Center, The Affiliated Hospital of Qingdao University, Qingdao, China; ^4^Department of Oncology, General Hospital of Ningxia Medical University, Yingchuan, China

**Keywords:** breast cancer, drug resistance, non-coding RNA, micro RNA, long-chain non-coding RNA

## Abstract

Breast cancer (BC) is one of the commonly occurring malignancies in females worldwide. Despite significant advances in therapeutics, the mortality and morbidity of BC still lead to low survival and poor prognosis due to the drug resistance. There are certain chemotherapeutic, endocrine, and target medicines often used for BC patients, including anthracyclines, taxanes, docetaxel, cisplatin, and fluorouracil. The drug resistance mechanisms of these medicines are complicated and have not been fully elucidated. It was reported that non-coding RNAs (ncRNAs), such as micro RNAs (miRNA), long-chain non-coding RNAs (lncRNAs), and circular RNAs (circRNAs) performed key roles in regulating tumor development and mediating therapy resistance. However, the mechanism of these ncRNAs in BC chemotherapeutic, endocrine, and targeted drug resistance was different. This review aims to reveal the mechanism and potential functions of ncRNAs in BC drug resistance and to highlight the ncRNAs as a novel target for achieving improved treatment outcomes for BC patients.

## Introduction

Breast cancer (BC), a complicated and heterogeneous disease which has high metastasis and recurrence rate, is a diverse hormone-dependent malignancy carcinoma and is leading in cancer mortality and morbidity globally. More than 20 million BC patients are newly diagnosed in women worldwide (1). Because of the heterogeneity of BC, drug resistance has become one of the major challenges. Although certain advances in research have been applied, the drug resistance of BC is still responsible for the poor prognosis and quite low survival (2). There are certain chemotherapeutic, endocrine, and targeted drugs available which have significantly improved the life quality and overall survival of patients, including anthracyclines, taxanes, cisplatin, and fluorouracil. For these therapeutic drugs, the mechanisms of drug resistance are complicated and have not been fully elucidated.

Non-coding RNAs (ncRNAs), including microRNAs (miRNAs), long-chain non-coding RNAs (LncRNAs), piRNAs, and circle RNAs (circRNAs), a group of RNAs which lack protein-coding regions, only account for about 1% of total genome RNA (3). Although these ncRNAs are less abundant, they exhibited essential performance in transcription, posttranscription, translation, and regulation of cellular processes and signaling pathways in the development and pathology of different cancer cells (4, 5). Besides, ncRNAs also have a significant influence on the exon gene coding *via* a different mechanism. The ability of ncRNAs to control gene expression makes them as targets or the key regulating genes for the tumor drug resistance (6).

Previous research reported that ncRNAs have the ability to modulate the sensitivity of cancer cell therapy. This ability contributed to the cancer cell drug resistance acquisition. This review summarizes the possible roles of ncRNAs in drug resistance following different mechanisms, highlighting the therapeutic and diagnostic application of ncRNAs for overcoming the BC resistance.

## ncRNAs and Anthracycline Chemoresistance

Anthracyclines are a group of antibiotics that are among the most active chemotherapeutic agents. The commonly used anthracycline antibiotics include doxorubicin, daunorubicin, and epirubicin (7). Anthracyclines exhibited a critical role in treating BC and can be used at all BC stages (8). Unfortunately, these agents also exhibited a well-recognized cardiotoxic profile that limits its clinical application (9). Several studies reported the mechanism of the chemoresistance of anthracycline and showed that ncRNAs exhibited high possibility in regulating BC resistance.

### miRNA and Anthracycline Chemoresistance

Chen et al. (10) found that the expression of miR-200c was related to doxorubicin-resistant BC. Upregulation of miR-200c could improve the epirubicin chemoselectivity and is also capable of decreasing the expression of P-glycoprotein (P-gp) and multidrug resistance mRNA in the human MCF-7/ADR cell line. Kopp et al. (11) found that decreased miR-200c expression in doxorubicin-resistant epithelial BC cell line BT474 could make the cells display the mesenchymal cell characteristics. Inhibition and overexpression miR-200c in the cells enhanced its resistance to doxorubicin treatment. Li et al. (12) and Park et al. (13) reported that miR-34a was down-expressed in MCF-7/ADR cells as compared to MCF-7 cells. Overexpression of miR-34a could increase the sensitivity of MCF-7/ADR cells to doxorubicin treatment by targeting NOTCH1. Zheng et al. (14) found that miR-181b performed the function of oncogenes during the development of BC and chemoresistance. Zhao et al. (15) found that downregulation of the miR-302S family genes miR-302d, miR-302c, miR-302b, and miR-302a could increase P-gp expression and enhance the chemoresistance of MCF-7/ADR cells. The enhanced expression of miR-302 facilitated the ADM agglomeration at extracellular and increased the sensitivity of BC cells for ADM. Spindlin1 (SPIN1) is an extremely expressed protein in various cancer types and is also associated with tumor development and genesis. Chen et al. (16) found that SPIN1 was a novel target of the miR-148/152 family; upregulation of miR-148/152 inhibited the SPIN1 expression and induced UGT2B4, CYP2C8, UGT2B17, and ABCB4 increase. The increase of UGT2B4, CYP2C8, UGT2B17, and ABCB4 is thereby involved in drug metabolism and transport along with enhancement in ADM resistance in BC. Hu et al. (17) reported the abrupt expression of ABCC4 and miR-124-3p in BC and MCF-7/ADR cells. After inhibiting the expression of ABCC4 and miR-124-3p, the sensitivity of the cells toward ADM was significantly increased. Doxorubicin was located in the cytoplasm rather than the nuclei of resistant cells due to the increased nuclear expression of MDR1/P-gp. Bao et al. (18) found that overexpression of miR-298 could inhibit P-gp and increase the P-gp nuclear accumulation and cytotoxicity in doxorubicin-resistant BC cells. The results suggested that miR-298 directly affects P-gp expression and influenced metastatic BC chemoresistance. Shen et al. (19) reported that miR-29a could play an important role in ADM resistance by inhibiting the PTEN/AKT/GSK3β signaling pathway in BC cells.

Miao et al. (20) revealed that miR-130b induced BC cell chemoresistance and promoted its proliferation through targeting PTEN and PI3K/Akt signaling pathway. Besides, the Wang group showed that miR-222 was capable of decreasing the sensitivity of BC cells to ADM through the PTEN/Akt/p27kip1 signaling pathway (21). The major cause of chemoresistance in BC was the overexpression of multidrug resistance-associated protein 1 (MRP1). Gao et al. (22) found that miR-145 could directly target MRP1 3′-untranslated regions and suppression of MRP1 expression. Overexpression of miR-145 could inhibit MRP1 expression and improve the extracellular doxorubicin accumulation. Jiang et al. (23) illustrated that the EMT-related chemoresistance in BC cells was mediated by miR-489. In their report, the EMT features and chemoresistance of ADM resistance cells (MCF-7/ADM) were reversed by overexpression of miR-489 through targeting Smad3. Meanwhile, Hu et al. (24) observed that overexpression of miR-760 increased the sensitivity of BC cells for certain anticancer agents *via* improved EMT transfer. The results proved that miR-760 was capable of modulating the chemoresistance of BC cells through EMT. Zhang et al. (25) explored the role of miRNA-192-5p in doxorubicin-resistant BC cells. They found that miR-192-5p overexpression was capable of activating JNK, augmenting Bad and caspase9, and suppressing the expression of Bcl-2 and PPIA. Zhao et al. (26) reported the correlation between miR-221 expression and the status of the hormone receptor (HR). In the research, they found that the patients with an increased miR-221 level in the plasma were considered to be HR-negative, and miR-221 can be a biomarker for evaluating the sensitivity of BC patients who previously received neoadjuvant chemotherapy.

### LncRNAs in Anthracycline Chemoresistance

LncRNAs comprise a group of over 200 nucleotides containing non-coding RNA molecules, while microRNAs include almost 21 nucleotides containing non-coding regulatory transcripts. It was reported that lncRNAs are involved in various drug resistance- and carcinogenesis-related genomics and cellular processes. The significance of lncRNAs was also discussed in the BC resistance against multiple drugs. For example, the expression of lnc00518 and multidrug resistance protein 1 (MRP1) was observed in MDR breast cells (MCF-7/ADR) compared with the normal MCF-7 line (27). lnc00518 was capable of reducing the apoptosis through inhibiting the miR-199a/MRP1 axis and increasing the resistance of MCF-7/ADR cells to VCR and ADM. Liang et al. (28) suggested that overexpression of lncLINP1 was positively related to the proliferation, chemoresistance, and metastasis of BC cells. Knockdown of LINP1 promoted BC cell metastasis and increased its resistance to 5-Fu by decreasing the effects of P53. Yao et al. (29) found that lncRNA NONHSAT101069 acted as ceRNA with miR-129-5p and targeted Twist1 in BC cells. The expression of lncRNA NONHSAT101069 promoted the resistance of BC cells to epirubicin and induced the cell EMT and migration process through the lncRNA NONHSAT101069/miR-129-5p/Twist1 axis. Gooding et al. (30) reported that lncRNABORG promoted the triple-negative BC (TNBC) cell chemoresistance to doxorubicin by activating the NF-κB signaling pathway. Chen et al. (31) found that LncRNAGAS5 significantly reversed the BC cell drug resistance by suppressing the Wnt/β-catenin signaling pathway through the miR-221-3p/DKK2 axis (**Table 1**, **Supplement Figure 1**).

**Table 1:** Breast cancer anthracycline chemoresistance-related ncRNAs.

ncRNA	Drugs	Function	Targets/mechanisms	References
miR-200c	Doxorubicin	Sensitivity	Inhibition P-gp	Chen et al. (10) and Kopp et al. (11)
miR-34a	Adriamycin	Sensitivity	Inhibition Notch1	Li et al. (12) and Park et al. (13)
miR-302a/b/c/d	Adriamycin	Sensitivity	Activation P-gp MAPK/ERK	Zhao et al. (15)
miR-148/152	Adriamycin	Resistance	Inhibition SPIN1	Chen et al. (16)
miR-124-3p	Adriamycin	Sensitivity	Inhibition ABCC4	Hu et al. (17)
miR-298	Adriamycin	Resistance	Inhibition P-gp	Bao et al. (18)
miR-29a	Adriamycin	Resistance	Inhibition PTEN/AKT/GSK3β	Shen et al. (19)
miR-130b	Adriamycin	Resistance	Inhibition PI3K/AKT	Miao et al. (20)
miR-222	Adriamycin	Resistance	Inhibition PTEN/AKT/p27 ^KIP1^	Wang et al. (21)
miR-145	Doxorubicin	Sensitivity	Inhibition MRP1	Gao et al. (22)
miR-489	Adriamycin	Sensitivity	Inhibition EMT/Smad3	Jiang et al. (23)
miR-760	Doxorubicin	Resistance	Inhibition EMT/Nanog	Hu et al. (24)
miR-192-5p	Doxorubicin	Sensitivity	Activation JNK/Bad/Caspase9, inhibition Bcl-2/PPIA	Zhang et al. (25)
miR-221	Adriamycin	Sensitivity	Inhibition hormone receptor(HR)	Zhao et al. (26)
LncRNA-00518	Adriamycin	Resistance	Inhibition miR-199a/MRP1 axis	Chang et al. (27)
LncRNA-LINP1	Doxorubicin	Resistance	Inhibition P53	Liang et al. (28)
LncRNA-NONHSAT101069	Epirubicin	Resistance	Inhibition miR-129-5p/Twist1/EMT	Yao et al. (29)
LncRNA-BORG	Doxorubicin	Resistance	Activation NF-κB signaling pathway	Gooding et al. (30)
LncRNA-GAS5	Adriamycin	Resistance	Inhibition Wnt/β-Catenin	Chen et al. (31)

## ncRNA and Tamoxifen Resistance

Tamoxifen is the most commonly used chemotherapeutic agent in the treatment of BC, specifically the estrogen receptor (ER)-positive BC subtype (32). Tamoxifen is considered a pioneering drug due to its ubiquitous use, cost-effectiveness, lifesaving properties, and being devoid of major side effects in the majority of BC patients (33). The ER-positive BC accounted for more than 70% of all breast cancers (34). However, ER-positive patients with metastatic disease poorly responded to tamoxifen therapy, and often with increased dose- and time-developed resistance to tamoxifen (35). For most ER-positive/progesterone receptor (PR)-negative BC subtypes, the 5-year survival was still quite low (∼20%) (36). The increased intrinsic and extrinsic factors are also responsible for the resistance toward chemotherapy in BC cells (37). It is quite urgent to better understand the mechanism of tamoxifen resistance and developed new therapies for BC.

### miRNAs in Tamoxifen Resistance

Tamoxifen is often used for ER-positive BC treatment. However, the tumor cells could develop resistance to tamoxifen and limit its application. Gene regulation by miRNAs often leads to activation or dysregulation of various pathways responsible for the development of drug resistance (38). Different miRNAs have been reported to be potential indicators for drug sensitivity in BC cell lines (39). Many miRNAs associated with tamoxifen resistance have been identified and offer new targets for BC therapy (40). Gao et al. (41) reported that the decrease of miR-200b and miR-200c reduced the expression of c-MYB and therefore elevated EMT marker vimentin and ZEB1/2 in tamoxifen-resistant ER-positive MCF-7 cells. Epiregulin (EREG), an EGFR agonist, plays a vital role in enhancing the process of glycolysis by activation of EGFR signaling and its downstream glycolytic genes in tamoxifen-resistant BC cells (42, 43). He et al. (44) found that in tamoxifen-resistant BC cells, EREG as a target of miR-186-3p and miR-186-3p is involved in BC cell resistance to tamoxifen. In HER2-positive tamoxifen-resistant primary human breast tumors, miR-221 and miR-222 directly targeted p27Kip1 and are responsible for increasing cell apoptosis upon exposure with tamoxifen (45). Li et al. (46) reported that miR-449a performed its function by targeting ADAM22 and took part in the underlying mechanism of tamoxifen resistance in BC. Another research reported that overexpression of miR-451a promoted the sensitivity of tamoxifen in BC by regulating the macrophage migration inhibitory factor and 14-3-3ζ ERα (47, 48). Ye et al. (49) examined the differential miRNA expression profiles between tamoxifen-resistant (MCF-7C and MCF-7T) and tamoxifen-sensitive (MCF-7) BC cell lines and showed that miR-21, miR-27a, miR-146a, miR-148a, and miR-34a performed a major role in tamoxifen resistance in BC.

### LncRNAs in Tamoxifen Resistance

Approximately 70% of BC patients have luminal A/ER-positive (ER+) BC which consists of genes with low proliferation rates and low levels of HER2 (50). A previous study showed that several lncRNAs demonstrated important roles in tamoxifen resistance (51). Li et al. (52) revealed that long non-coding RNA UCA1 conferred tamoxifen resistance in BC endocrine therapy through activation of the EZH2/p21 axis and the PI3K/AKT signaling pathway. Liu et al. (53) reported that lncRNA CYTOR has the function of promoting tamoxifen resistance in BC cells *via* sponging miR-125a-5p. Xue et al. (54) observed that LncRNA HOTAIR was upregulated in tamoxifen-resistant breast cancer tissues compared to their primary counterparts. Overexpression of HOTAIR increased the proliferation BC cells and enhanced their tamoxifen resistance. Ma et al. (55) determined that the expression of lncRNA LINP1 (non-homologous end joining pathway 1) was increased in tamoxifen-resistant BC cells. Knockdown of lncRNA LINP1 significantly attenuated the tamoxifen resistance *in vitro* and *in vivo*. lncRNA HOTAIRM1 has been proved to be involved in myelopoiesis as well as transcription regulation of HOXA genes in embryonic stem cells. In BC cells, lncRNA HOTAIRM1 and HOXA1 are upregulated in tamoxifen-resistant MCF7 (TAMR) cells, and the knockdown of lncRNA HOTAIRM1 downregulated the HOXA1 expression and restored the sensitivity to tamoxifen (56). Cyclin D1 is one of the most important cancer proteins that drive cancer cell proliferation and associate with tamoxifen resistance in BC. Shi et al. (57) proved that LncRNA DILA1 inhibits Cyclin D1 degradation and contributes to tamoxifen resistance in breast cancer. Qu et al. (58) reported that lncRNA BLACAT1 was significantly upregulated in tamoxifen-resistant BC cells MCF-7/TR and T47D/TR, and knockdown of lncRNA BLACAT1 reduced the tamoxifen resistance in the cells. Further study revealed that lncRNA BLACAT1 induced tamoxifen resistance through regulating the miR-503/Bcl-2 axis in BC. Ma et al. (59) reported that LncRNA DSCAM-AS1 enhanced BC cell tamoxifen resistance through acting as a sponge of miR-137. Xu et al. (60) found that tamoxifen-resistant BC cell-derived exosomes contain lncRNA urothelial cancer-associated 1 (UCA1), and the expression of lncRNA UCA1 increased tamoxifen resistance in BC. LncRNA UCA1 was also found to be involved in causing tamoxifen resistance in BC cell lines MCF7 and T47D by activating the Wnt/β-Catenin signaling pathway (61) and mTOR signaling pathway (62). Shi et al. (63) identified that lncRNA ADAMTS9-AS2 has a lower expression in BC tissues and tamoxifen-resistant BC cells. A low expression of lncRNA ADAMTS9-AS2 inhibited PTEN expression and enhanced tamoxifen resistance through targeting miRNA-130a-5p. Zhang et al. (64) revealed that downregulation of lncRNA ROR inhibited the BC cell EMT and enhanced the cell sensibility to tamoxifen through increasing miR-205 expression.

### CircRNAs in Tamoxifen Resistance

CircRNAs are a group of ncRNAs which contributed to the gene regulation by competing the combination with endogenous RNA (ceRNA) mechanisms (65). CircRNAs often serve as transcription regulators, acting as microRNA sponges and expressing peptides under rare circumstances and sequestering RNA-binding proteins (RBPs) (66). Sang et al. (67) found that the expression of hsa_circ_0025202 enhanced tamoxifen efficacy and inhibited the progression of BC cells *via* regulating the miR-182-5p/FOXO3a axis. Liang et al. (68) reported that knockdown of CircBMPR2 promoted tamoxifen resistance and inhibited apoptosis of BC cells *via* the circBMPR2/miR-553/USP4 axis. Hu et al. (69) showed that circ_UBE2D2 isolated from exosomes enhanced the resistance of BC cells to tamoxifen by binding to miR-200a-3p. Uhr et al. (70) revealed that miR-7 is connected with tamoxifen treatment outcomes in an adjuvant hormone-naïve cohort, and circRNA CDR1-AS regulated miR-7 function in BC. However, circRNA CDR1-AS has negative relevant outcomes in the cohort (**Table 2**, **Supplement Figure 2**).

**Table 2:** Breast cancer tamoxifen chemoresistance-related ncRNAs.

ncRNA	Drugs	Function	Targets/mechanisms	References
miR-200b/c	Tamoxifen	Sensitivity	Activation of vimentin/ZEB/EMT	Gao et al. (41)
miR-186-3p	Tamoxifen	Resistance	Activation of EREG/EGFR	He et al. (44)
miR-221/222	Tamoxifen	Resistance	Inhibition of p27^Kip1^	Miller et al. (45)
miR-449a	Tamoxifen	Sensitivity	Inhibition of ADAM22	Li et al. (46)
miR-451a	Tamoxifen	Sensitivity	Inhibition of MIF	Liu and Liu et al. (47, 48)
lncRNA-UCA1	Tamoxifen	Resistance	Activation of PI3K/AKT	Li et al. (52)
lncRNA-CYTOR	Tamoxifen	Resistance	Activation of SRF and Hippo signaling pathway	Liu et al. (53)
lncRNA-HOTAIR	Tamoxifen	Resistance	Activation of ER signaling	Xue et al. (54)
lncRNA-LINP1	Tamoxifen	Sensitivity	Inhibition of ER and EMT	Ma et al. (55)
lncRNA-HOTAIRM1	Tamoxifen	Resistance	Inhibition of HOXA1	Kim et al. (56)
lncRNA-DILA1	Tamoxifen	Sensitivity	Inhibition of Cyclin D1	Shi et al. (57)
lncRNA-BLACAT1	Tamoxifen	Resistance	Activation of miR-503/Bcl-2 axis	Qu et al. (58)
lncRNA-DSCAM-AS1	Tamoxifen	Resistance	Activation of EPS8	Ma et al. (59)
lncRNA-UCA1	Tamoxifen	Resistance		Xu et al. (60)
lncRNA-UCA1	Tamoxifen	Resistance	Activation of Wnt/beta-Catenin signaling pathway	Liu et al. (61)
lncRNA-UCA1	Tamoxifen	Resistance	Inhibition of mTOR signaling pathway	Wu et al. (62)
lncRNA-ADAMTS9-AS2	Tamoxifen	Sensitivity	Inhibition of PTEN	Shi et al. (63)
lncRNA-ROR	Tamoxifen	Resistance	Inhibition of EMT	Zhang et al. (64)
circRNA-0025202	Tamoxifen	Sensitivity	Inhibition of FOXA3a	Sang et al. (67)
circRNA-BMPR2	Tamoxifen	Sensitivity	Inhibition of the miR-553/USP4 axis	Liang et al. (68)
circRNA-UBE2D2	Tamoxifen	Resistance	Inhibited of miR-200a-3p	Hu et al. (69)
circRNA-CDR1-AS	Tamoxifen	Resistance	Inhibition of hsa-miR-7	Uhr et al. (70)

## ncRNAs and Taxane resistance

Taxanes are an important class of antineoplastic agents often used for treatment of a wide variety of cancers. Paclitaxel and docetaxel are the most commonly used taxanes, which elicit immediate hypersensitivity reactions (HSRs) in 5% to 10% of patients (71). Almost all patients that experience HSRs can be safely reexposed to taxanes. Taxanes not only strengthen BC treatment but also are capable of developing resistance following mortality and metastatic disease (72). Taxanes are cytotoxic because they inhibit the depolymerization of tubulin microtubules and affect the process of mitosis in the M or G1 phase. Furthermore, it is also reported that the antineoplastic activity of taxanes is significantly involved in certain biological processes including angiogenesis, apoptosis, cell motility, invasiveness, and metalloproteinase production (73). Triple-negative breast cancer (TNBC) is a heterogeneous disease with various prognoses and chemosensitivity profiles, and the standard therapy includes the mainstay treatment with anthracyclines and taxanes (74). Although there have been many studies for exploring the cause of taxane resistance in BC, the mechanism of the process is still unknown. ncRNAs could regulate the expression of drug resistance gene and thereby influence the BC cell progression and development of chemotherapy resistance (75).

### ncRNAs in Paclitaxel Resistance

#### miRNAs in Paclitaxel Resistance

Various miRNAs have been reported to be related to different cancers (76). In BC cells, Lin28/let-7 is related to paclitaxel resistance and the Lin28 miRNA level is intensely improved in tissues of tumors following neoadjuvant chemotherapy (77). Lin28 has conferred specified cancer stem cells to BC cells and help the cells to gain the properties of “stemness” so that they can escape from the effect of chemotherapy. Overexpression of Lin28 is capable of inducing Rb and p21 expression and decreasing the level of let-7a (78). Tsang also reported that let-7a directly targeted caspase 3 and promoted the resistance in paclitaxel-induced apoptosis (79). Tao et al. (80) proved that downregulation of let-7f was associated with its target thrombospondin-1 (TSP-1) and thus influenced the cell sensibility to paclitaxel in MCF-7 cells. With the help of the miRNA array, Zhou et al. (81) observed the upregulation of miR-125b, miR-221, miR-222, and miR-923 in paclitaxel-resistant BC cells. They also proved that miR-125b can inhibit the paclitaxel-induced apoptosis and cytotoxicity by suppressing the expression of pro-apoptotic Bcl2 antagonist killer 1 (BAK1) in BC cells.

Another miRNA involved in paclitaxel resistance was miR-520h; the increased expression of miR-520h was correlated with negligible prognosis and lymph node metastasis in human BC patients. The expression of miR-520h promoted paclitaxel resistance of human breast cancer cells through suppressing death-associated protein kinase 2 (DAPK2) expression and protecting the cells from paclitaxel-induced apoptosis (82). Gu et al. (83) reported that miR-451 possesses a significant influence to the sensibility of neoadjuvant chemotherapy by inhibiting the expression of Bcl-2 and the process of apoptosis induced by paclitaxel. The luminal A subtype was a special type of BC which exhibited ER^+^/PR^+^ and HER2 (84). In luminal A BC cells, miR100 proved to sensitize the cells to paclitaxel treatment in part by targeting the mTOR signaling pathway. The results showed that microRNA 100 plays important roles for luminal A subtype BC cell resistance to paclitaxel (85). In TNBC cells, overexpression of miR-18a was reported to reduce the expression of DICER and enhance autophagy and paclitaxel resistance by inhibiting the mTOR signaling pathway (86). Liu et al. (87) illustrated that the expression of miR-101 in TNBC cells significantly inhibited the effects of tumorigenesis *in vivo* and growth and apoptosis *in vitro*. Besides, miR-101 also increased paclitaxel sensitivity by suppressing myeloid cell leukemia-1 (MCL-1) expression in TNBC cells.

#### LncRNAs in Paclitaxel Resistance

Arun et al. (88) reviewed the function and mechanism of lnc-MALAT1 (MALAT1) in BC and proved that the patients with elevated MALAT1 showed worse prognosis. Yu et al. (89) used MCF-7/Tax (taxane-resistant MCF-7 cells) and MCF-7/Adr (adriamycin-resistant MCF-7 cells) cells as research objects. They found that MALAT1 exhibited a significantly high level in the cells, and knockdown of MALAT1 decreased the sensitivity of the cells to taxane and adriamycin. Zheng et al. (90) found that long non-coding RNA CASC2 (CASC2) regulated the expression of miR-18a-5p/CDK19 and activated paclitaxel resistance in BC. Thus, they highlighted the significance of the CASC2/miR-18a-5p/CDK19 axis in the chemoresistance of BC and provided potential aims to improve the chemotherapy of BC.

Unlike ER^+^ and HER2^+^ BC, TNBC patients are primarily treated with chemotherapy. Paclitaxel is the first-line taxane-based chemotherapeutic agent that is used for the treatment of TNBC patients (91). Si et al. showed that lncRNA H19 was one of the downstream target molecules of ERα. Altered ERα expression could change H19 levels and modulate the apoptosis response to chemotherapy in BC cells. They also suggested that the ERα-H19-BIK signaling axis plays an important role in promoting chemoresistance for Erα+ BC to paclitaxel (92). Raveh et al. (93) found that lncRNA-H19 was elevated in TNBC paclitaxel-resistant cell lines compared to parental cells. LncRNA-H19 was highly expressed during embryonic development but decreased after birth, specifically in mammary tissue. Knockdown of lncRNA-H19 in paclitaxel-resistant TNBC cell lines increased paclitaxel sensitivity by reducing p-AKT (Ser473) and decreasing the apoptotic rate (94). Chen et al. (95) identified that Linc00839 was localized in the nucleus and upregulated in chemoresistant BC cells and tissues. The expression of Linc00839 was activated by Myc and promoted proliferation and chemoresistance in breast cancer through binding with Lin28B *via* activation of the PI3K/AKT signaling pathway.

#### CircRNA in Paclitaxel Resistance

circRNAs also play vital roles in the paclitaxel resistance of BC cells. Ma et al. (96) identified that circular RNA angiomotin-like 1 (circAMOTL1) has high correlations with paclitaxel resistance in BC cells. circAMOTL1 regulated the AKT pathway and facilitated the anti-apoptotic protein expression which led to paclitaxel resistance in BC cells. Yang et al. (97) reported that circ-ABCB10 bound with let-7a-5p and promoted paclitaxel sensitivity and apoptosis while suppressing invasion and autophagy of paclitaxel-resistant BC cells. Zang et al. (98) proved that circ-RNF111 was upregulated in paclitaxel-resistant BC tissues and cells. Knockdown of circ-RNF111 reduced the function of paclitaxel on BC cells. They further identified miR-140-5p as a target of circ-RNF111, and circ-RNF111 improved paclitaxel resistance of BC cells by upregulating E2F3 *via* sponging miR-140-5p (**Table 3**, **Supplement Figure 3**).

**Table 3:** Breast cancer paclitaxel chemoresistance-related ncRNAs.

ncRNA	Drugs	Function	Targets/mechanisms	References
Lin28	Paclitaxel	Resistance	Activation of p21 and Rb; inhibition of Let-7	Lv et al. (78)
Let-7a	Paclitaxel	Resistance	Inhibition of caspase-3	Tsang et al. (79)
mi-125b	Paclitaxel	Resistance	Inhibition of BAK1	Zhou et al. (81)
mi-520h	Paclitaxel	Resistance	Inhibition of DAPK2	Su et al. (82)
mi-451	Paclitaxel	Resistance	Inhibition of Bcl-2	Gu et al. (83)
mi-100	Paclitaxel	Sensitivity	Inhibition of the Mtor signaling pathway	Zhang et al. (85)
mi-18a	Paclitaxel	Resistance	Inhibition of the mTOR signaling pathway	Sha et al. (86)
mi-101	Paclitaxel	Sensitivity	Inhibition of MCL-1	Liu et al. (87)
LncRNA-CASC2	Paclitaxel	Resistance	Inhibition miR-18a-5p/CDK19	Zheng et al. (90)
LncRNA-H19	Paclitaxel	Resistance	Inhibition AKT/BIK	Si et al. (92) and Raveh et al. (93) and Han et al. (94)
LncRNA-00839	Paclitaxel	Resistance	Activation PI3K/AKT signaling pathway	Chen et al. (95)
CircRNA-ABCB10	Paclitaxel	Resistance	Inhibition of the let-7a-5p/DUSP7 axis	Yang et al. (97)
CircRNA-RNF111	Paclitaxel	Resistance	Inhibition of miR-140-5p/E2F3	Zang et al. (98)

### ncRNAs in Docetaxel Resistance

#### miRNAs in Docetaxel Resistance

Docetaxel (a semi-synthetic paclitaxel analog) was synthesized by the precursor obtained from the needles of the European yew. Paclitaxel and docetaxel both performed their function by inhibiting mitotic activity and suppressed the polymerization of microtubules (99). There are various miRNAs whose downregulation plays a vital role in BC cell docetaxel resistance (100, 101). For example, an *in vitro* study revealed that the elevated level of miR-129-3p was interlinked with docetaxel resistance by directly inhibiting the apoptosis-associated protein eukaryotic translation initiation factor 4E (EIF4E). Downregulation of miR-141 resulted in a decrease of EIF4E/CP110 and provided an apoptosis-inducing effect (102). Another study revealed that miR-129-3p promoted docetaxel resistance of BC cells *via* inhibiting the expression of centriolar coiled-coil protein 110 (CP110) (103). In MCF-7 and MDA-MB-231 BC cell lines, an upregulation of miR-3646 was related to docetaxel resistance through activating the GSK-3β/β-catenin signaling pathway (104). Hu et al. (105) observed the expression of miR-663, and miR-452 was increased in docetaxel-resistant BC cell lines MDAMB-231 and MCF-7. MiR-452 contributed to the docetaxel resistance by inhibiting anaphase-promoting complex subunit 4 (APC4) expression, while overexpression of miR-663 caused the downregulation of heparin sulfate proteoglycan 2 (HSPG2) and induced BC cell chemoresistance (106). In extensive research, Kaslt et al. (107) conducted a microarray analysis of MDA-MB-231 and MCF-7 cell lines between docetaxel resistance and miRNA expression. The results showed that miR-141 and miR-34a were increased and miR-16, miR-7, miR-30a, miR-126, and miR-125a-5p were decreased in docetaxel resistance cells. Zhang et al. (108) also analyzed miRNA array and found that miR-139-5p was significantly downregulated in BC cells compared to vicinal typical tissue. The *in vitro* research revealed that miR-139-5p was capable of inhibiting BC cell growth and induced apoptosis by targeting Notch1 and hence decreasing the docetaxel resistance. Besides, miR-205 was reported to increase the sensitivity of MDA-231 and MCF-7 cells against docetaxel *via* inhibition of clonogenic capability and cell proliferation (109).

Xu et al. (110) found that miR-125a was downregulated in docetaxel-resistant BC cells, and overexpression of miR-125a enhanced the cells’ docetaxel sensitivity by suppressing the BRCA1 expression. The authors also observed that the level of miR-125a was decreased in the HER-2 and metastatic specimens of BC patients. The outcome provided a novel approach toward increased sensitivity of BC patients against docetaxel *via* overexpression of miR125a. Generally, a combined therapy of docetaxel plus adriamycin is used to treat metastatic and reoccurrence BC patients. However, development of drug resistance remains a latent problem, and miR-222 and miR-29a have been reported to increase in docetaxel plus adriamycin-resistant BC cell lines. Further research proved that the two miRNAs are potential inhibitors that altered the drug resistance and restored their sensitivity by targeting PTEN and activating the Akt/mTOR approach (111).

Exosomes, a group of 40–100-nm-nanosized vesicles that lived in the extracellular space of cells, perform as genome exchange vehicles between heterogeneous tumor cells. Exosomes are also capable of transferring drug resistance to desired cells through the miRNAs they contained. Chen et al. (112) reported that miR-23a, miR-1246, miR-1469, let-7b, miR-38 and miR-1915 were found in docetaxel-resistant cell exosomes, illustrating that these exosomes play important roles in the drug resistance cells.

#### LncRNAs in Docetaxel Resistance

Huang et al. (113) performed RNA sequencing and analyzed that mRNAs and lncRNAs contribute to docetaxel resistance in two docetaxel-resistant BC cell lines MCF7-RES and MDA-RES and their docetaxel-sensitive parental cell lines. Co-expression network and location analysis revealed that four lncRNAs might upregulate the expression of ABCB1 and influence the cells’ drug resistance. The author also identified the lncRNA EPB41L4A-AS2 (EPB41L4A antisense RNA 2) as a potential biomarker for docetaxel sensitivity BC cells. Shin et al. (114) revealed that the combination of cisplatin or taxol and NEAT1 (lncRNA nuclear paraspeckle assembly transcript 1) knockdown synergistically inhibited the cells’ sensitivity to the drug when compared with cisplatin or taxol alone. Overexpression of NEAT1 in cisplatin- and taxol-resistant TNBC cells indicated its function of chemoresistance in BC cells (**Table 4**, **Supplement Figure 4**).

**Table 4:** Breast cancer docetaxel chemoresistance-related ncRNAs.

ncRNA	Drugs	Function	Targets/mechanisms	References
miR-141	Docetaxel	Sensitivity	Activation of EIF4E/CP110	Yao et al. (102)
miR-129-3p	Docetaxel	Resistance	Inhibition of CP110	Zhang et al. (103)
miR-3646	Docetaxel	Resistance	Activation of the GSK-3β/β-catenin signaling pathway	Zhang et al. (104)
miR-452	Docetaxel	Resistance	Inhibition of APC4	Hu et al. (105)
miR-663	Docetaxel	Resistance	Inhibition of HSPG2	Hu et al. (106)
miR-139-5p	Docetaxel	Resistance	Inhibition of Notch1	Zhang et al. (108)
miR-125a-3p	Docetaxel	Sensitivity	Inhibition of BRCA1	Xu et al. (110)
miR-222/29a	Docetaxel	Resistance	Activation of Akt/mTOR	Zhong et al. (111)
LncRNA-EPB41L4A-AS2	Docetaxel	Sensitivity	Activation of ABCB1	Huang et al. (113)
LncRNA-NEAT1	Docetaxel	Resistance	Activation of Sox2/ALDH	Shin et al. (114)

## ncRNAs in 5-Fluorouracil resistance

5-Fluorouracil (5-FU) is a classic chemotherapeutic drug, and it has been extensively used to treat different cancers (115). However, patients often exhibited primary or acquired drug resistance during treatments. Although there are many advancements in bioresearch technologies in the past several decades, the molecular mechanisms of 5-FU resistance have not been completely clarified (116). ncRNAs as oncogenes or tumor suppressors often play a vital role in BC cells and contributed to 5-FU drug resistance (117).

### miRNAs in 5-FU Resistance

Nandy et al. (118) proved that microRNA-125a influences breast cancer stem cells by posttranscriptionally regulating the leukemia inhibitory factor (LIF) receptor gene expression *via* binding with its 3′-untranslated region (UTR), thus regulating the cells’ drug resistance to 5-FU through the Hippo signaling pathway. Zhang et al. (119) reported that the interaction between miR-508-5p and P-gp or ZNRD1 was responsible for 5-FU chemotherapeutic resistance. Moreover, Yin et al. (120) indicated that the direct repression of Bmi1 expression under the action of miR-200c and miR-203 could alter the Bmi1-mediated 5-FU resistance. Li et al. (121) illustrated that chemotherapeutics like 5-FU was involved in the suppression of miR-488 and which in turn activated the epidermal growth factor receptor (EGFR)/nuclear factor kappaB (NF-κB) signaling approach *via* targeting SATB1.

### LncRNAs in 5-FU Resistance

Several studies have been conducted to analyze the 5-FU resistance-related upregulation and downregulation of lncRNAs in BC. Redis et al. (122) reported that upregulation of Lnc-CCAT2 correlated with the sensitivity to 5-FU in BC cells. The ncRNA nuclear paraspeckle assembly transcript 1 (NEAT1) present at an elevated level in BC cells, especially in stage III–IV tumors vs. overexpression of lncRNA NEAT1, was related to the poor prognosis and metastasis of BC. Li et al. (123) conducted *in vitro* assays to understand the biological function of Lnc NEAT1 and observed that NEAT1 was involved in the sponging of miR-211 and induced BC cell resistance to 5-FU. The group of Chen and Hou reported that overexpression of LncROR was connected with BC cell EMT and therefore improved the cells’ invasion capability and resistance to 5-FU. This result suggests that upregulation of LncROR can be considered to be a potential drug resistance marker (124, 125). A recent research by Yao et al. (126) found that the ER stress induced by 5-FU could increase the expression of GRP78 in MCF-7 cells. GRP78 then regulated the expression of LncMIAT and AKT through upregulating Oct4, thereby increasing the BC cells’ resistance to 5-FU. The conclusion was that LncMIAT participated in BC cell resistance to 5-FU through the ER stress-mediated GRP78/Oct4/lncRNA MIAT/AKT pathway. Luo et al. (127) reported that overexpression of lncRNA SNORD3A specifically sensitizes breast cancer cells to 5-FU *via* enhancing UMPS expression. The SNORD3A-UMPS axis may serve as a potential biomarker and therapeutic target to improve the efficacy of 5-FU-based chemotherapy for BC patients.

### CircRNA in 5-FU Resistance

circRNAs are a class of ncRNA which have a circle structure. circRNAs have been discovered in various cancers and acted as either promoting tumorigenesis or inhibiting tumor progression (128). Regarding research on circRNAs and BC cell chemoresistance, only Yang et al. reported about circRNA CDR1as, implicating its function in regulating 5-FU sensitivity in BC cells (129). In the study, they found that circRNA CDR1as competitively inhibited miR-7 to regulate CCNE1 expression. The overexpression of circRNA CDR1as reversed the enhancement of 5-FU sensitivity in BC cells caused by overexpression of miR-7. The study proved that circRNA CDR1as regulated the sensitivity of 5-FU-resistant BC cells by inhibiting miR-7 to regulate CCNE1 (**Table 5**, **Supplement Figure 5**).

**Table 5:** Breast cancer fluorouracil chemoresistance related ncRNAs.

ncRNA	Drugs	Function	Targets/mechanisms	References
miR-125a	Fluorouracil	Resistance	Inhibition LIF/Hippo signaling pathway	Nandy et al. (118)
miR-508-5p	Fluorouracil	Resistance	Inhibition P-gp or ZNRD1	Zhang et al. (119)
miR-200/203	Fluorouracil	Sensitivity	Inhibition P53/Bmi1	Yin et al. (120)
miR-448	Fluorouracil	Resistance	Inhibition EMT/NFkB	Li et al. (121)
LncRNA-NEAT1	Fluorouracil	Resistance	Inhibition miR-211/HMGA2	Li et al. (123)
LncRNA-RoR	Fluorouracil	Resistance	Activation EMT	Chen et al. (124)
LncRNA-ROR	Fluorouracil	Resistance		Hou et al. (125)
LncRNA-MIAT	Fluorouracil	Resistance	Activation GRP78/OCT4/AKT pathway	Yao et al. (126)
LncRNA-SNORD3A	Fluorouracil	Sensitivity	Activation UMPS	Luo et al. (127)
Circ-CDR1as	Fluorouracil	Resistance	Inhibition miR-7/CCNE1	Yang et al. (129)

## ncRNAs in trastuzumab resistance

The human epidermal growth factor receptor 2 (HER-2) is often used to classify the BC patients with overexpression (known as HER-2 positive) or not (HER-2 negative) (130). There is a high correlation between HER-2 upregulation and BC metastasis as well as poor prognosis (131). Trastuzumab (TRS), a HER-2-targeting humanized monoclonal antibody, is a selective treatment that targets HER-2 (132). ncRNA provides a comprehensive understanding of their mechanism of action and function and crucial contribution in regulating BC drug resistance and metastasis (133).

### miRNAs in Trastuzumab Resistance

To validate the mechanism of miRNAs in BC trastuzumab resistance, several studies were conducted *in vivo* and *in vitro*. Gong and De Mattos et al. (134, 135) found that upregulation of miR-21 significantly correlated with BC resistance to trastuzumab by activation of PTEN, inhibition of AKT, and sustenance of EMT. However, Nielsen et al. (136) reported that the expression of miR-21 in primary breast cancer may not predict its resistance to adjuvant trastuzumab treatment. Ye et al. (137) proved that miR-221 promoted HER-2-positive BC against trastuzumab through suppressing PTEN expression. Besides, the circulating level of miR-210 in plasma was found to be correlated with HER-2-positive BC patients who are trastuzumab resistant, indicating that plasma miR-210 could serve as a predictive biomarker in surveillance of the therapeutic responsiveness (138). Bai et al. (139) found that miR-200c counteracts trastuzumab resistance and metastasis by inhibiting ZNF217 and ZEB1 and TGF-beta signaling pathway expression in BC. Ye and Ma et al. (140, 141) reported that downregulation of miR-5423p and miR-375 contributed to induction of TRS resistance in HER-2-positive breast cancer through inhibition of IGF1R and activation of the PI3K/AKT signaling pathway. Corcoran et al. (142) proved that downregulation of miR-630 tightly connected with HER-2-targeting drugs in HER-2-overexpressing BC by inhibition of IGF1R. Venturutti et al. (143) found that mir-16 was upregulated in HER-2-positive breast cancer and mir-16 mediated trastuzumab and lapatinib response in ErbB-2-positive breast cancer *via* its novel targets CCNJ and FUBP1. Huynh et al. (144) reported that microRNA-7 reversed TRS resistance by HER-2 Delta16 and multiple oncogenic pathways in breast cancer cells.

### LncRNAs in Trastuzumab Resistance

LncRNA, as a group of ncRNA, also played an important role in HER2^+^ BC trastuzumab resistance, but its contribution to BC resistance is still unclear. Trastuzumab was considered to be the first-line therapy drug to treat advanced HER2^+^ BC (145). It was reported that LncRNA-SNHG14 was responsible for mediating trastuzumab *via* extracellular exosomes of tumor cells. Exosomal lncRNA-SNHG14 activated the Bcl-2/Bax apoptosis signaling pathway and induced resistance against trastuzumab in BC cells. When treating the cells with trastuzumab-resistant cell-derived exosomes, the cell apoptosis and death were remarkably decreased (146). Dong et al. (147) reported that lncRNA AGAP2-AS1 promoted the growth of BC and trastuzumab resistance by upregulation of MyD88 expression by activating the NF-κB signaling approach. Based on the microarray analysis, Shi et al. (148) observed that lncRNA-ATB was elevated in five trastuzumab-resistant BC patients. Further study revealed that lncRNA-ATB promoted trastuzumab resistance *via* activating the EMT and TGF-β signaling pathway in BC cells. Li et al. (149) reported the significant downregulation of LncGAS5-activated miR21 and mTOR signaling pathway in trastuzumab-resistant SKBR-3 cells and trastuzumab-resistant BC patients. Han et al. (150) observed that lncZNF649-AS1 was highly expressed in trastuzumab-resistant cells compared to sensitive cells. lncZNF649-AS1 was upregulated by H3K27ac modification in the presence of trastuzumab treatment. Knockdown of ZNF649-AS1 reversed trastuzumab resistance *via* modulating ATG5 expression. Chen et al (151) found that LncRNA HOTAIR was highly expressed in trastuzumab-resistant cell line SK-BR-3-TR, and blocking of HOTAIR expression restores the sensitivity. LncRNA HOTAIR is involved in BC cell trastuzumab resistance *via* epigenetic modification of methylation in PTEN and therefore activation of the TGF-β signaling pathway (**Table 6**, **Supplement Figure 6**).

**Table 6:** Breast cancer trastuzumab chemoresistance-related ncRNAs.

ncRNA	Drugs	Function	Targets/mechanisms	References
miR-21	Trastuzumab	Resistance	Activation of PTEN Inhibition of AKT and NF-κB	Gong and De Mattos et al. (134, 135)
miR-221	Trastuzumab	Resistance	Inhibition of PTEN	Ye et al. (137)
miR-200c	Trastuzumab	Resistance	Inhibition of ZNF217/ZEB1/TGF-β signaling pathway	Bai et al. (139)
miR-375	Trastuzumab	Sensitivity	Inhibition of IGF1R,	Ye et al. (140)
miR-542-3p	Trastuzumab	Sensitivity	Activation of PI3K/AKT	Ma et al. (141)
miR-630	Trastuzumab	Sensitivity	Inhibition of IGF1R	Corcoran et al. (142)
miR-16	Trastuzumab	Sensitivity	Inhibition of CCNJ and FUBP1	Venturutti et al. (143)
miR-7	Trastuzumab	Resistance	Inhibition of EGFR	Huynh et al. (144)
LncRNA-SNHG14	Trastuzumab	Sensitivity	Activation of Bcl-2/Bax	Dong et al. (146)
LncRNA-AGAP2-AS1	Trastuzumab	Resistance	Activation of MyD88/NF-κB signaling pathway	Dong et al. (147)
LncRNA-ATB	Trastuzumab	Resistance	Activation of EMT/TGF-β signaling	Shi et al. (148)
LncRNA-GAS5	Trastuzumab	Sensitivity	Activation of miR21/mTOR signaling pathway	Li et al. (149)
LncRNA-ZNF649-AS1	Trastuzumab	Resistance	Activation of ATG5/PTBP1	Han et al. (150)
LncRNA-HOTAIR	Trastuzumab	Resistance	Activation of TGF-β signaling pathway	Chen et al. (151)

## Conclusion

Drug resistance is one of the main causes of BC therapy failure in clinical settings. It is also a complex process involving multiple factors, multiple steps, and multiple genes. Despite a number of novel agents that have been developed, the truly efficient options with minimal adverse effects for BC treatment remain limited. In this article, we summarized the mechanisms of ncRNAs in BC drug resistance, including chemotherapeutic, endocrine, and targeted drug resistance. Based on the reports, the molecular mechanisms of ncRNAs involved in BC drug resistance include 1) ncRNAs as a target gene of drugs and influencing its effects, 2) ncRNAs acting as ceRNAs to modulate BC cell sensitivity and drug resistance, 3) ncRNAs regulating cancer cell apoptosis and cell cycle transfer, and 4) ncRNAs inducing BC cell drug resistance through NF-KB, mTOR, and Wnt/β-catenin signaling pathways.

Even though there are many studies about the mechanism of ncRNAs in BC drug resistance, some of them even highlighted ncRNAs as a novel target for achieving improved treatment outcomes for BC patients. The mechanism of ncRNA networks regulating drug resistance and the selection of key targets from numerous candidate ncRNAs remain challenging. Besides, despite that most of current studies used human BC cell lines cultured *in vitro*, there still lack clinical studies to explore the mechanism of ncRNAs in BC drug resistance.

Although we reviewed the most research of ncRNA in BC drug resistance in this article, the details of mechanism still need further exploring. With the development of technology and the new research elucidates, we believe that targeting ncRNAs could be a novel strategy for achieving improved treatment outcomes for BC patients in the future.

## Author Contributions

L-bW contributed to the conception and design of this study. The acquisition of data was carried out by S-hL and C-yY. The analysis of data was carried by J-hT. All authors contributed to the article and approved the submitted version.

## Funding

This study was funded by the Science and Technology Projects of Ningxia Key R&D Programs (No. 2019BFH02012, 2021BEG03089); The National Natural Science Foundation of China (No. 81860470); The Ningxia High Level Science and Technology Innovation Leading Talent Project (No. KJT2019003); The Ningxia Biochip Technology Research and Development Innovation Team (No. 2019-18); The Scientific Research Platform Open Project of the General Hospital of Ningxia Medical University (No. 2020-146); The Science Research Project of Ningxia Medical University (No.XM2018099); The Science Research Project of the General Hospital of Ningxia Medical University (No. XM2020159). The First-Class Discipline Construction Project of NingXia Medical University and the School of Clinical Medicine (No. NXYLXK2017A05).

## Conflict of Interest

The authors declare that the research was conducted in the absence of any commercial or financial relationships that could be construed as a potential conflict of interest.

## Publisher’s Note

All claims expressed in this article are solely those of the authors and do not necessarily represent those of their affiliated organizations, or those of the publisher, the editors and the reviewers. Any product that may be evaluated in this article, or claim that may be made by its manufacturer, is not guaranteed or endorsed by the publisher.

## Supplementary Material

Supplementary Figure 1 The pattern diagram of ncRNAs and Anthracyclines chemoresistance.

Supplementary Figure 2 The pattern diagram of ncRNAs and Tamoxifen chemoresistance.

Supplementary Figure 3 The pattern diagram of ncRNAs and Paclitaxel chemoresistance.

Supplementary Figure 4 The pattern diagram of ncRNAs and Docetaxel chemoresistance.

Supplementary Figure 5 The pattern diagram of ncRNAs and Fluorouracil chemoresistance.

Supplementary Figure 6 The pattern diagram of ncRNAs and Trastuzumab chemoresistance.
