# Supplementary figures and images for: The Role of Non-Coding RNAs in Breast Cancer Drug Resistance
Source: Front Oncol. 2021 Sep 13;11:702082. doi: 10.3389/fonc.2021.702082 (PMC8473733; doi:10.3389/fonc.2021.702082)

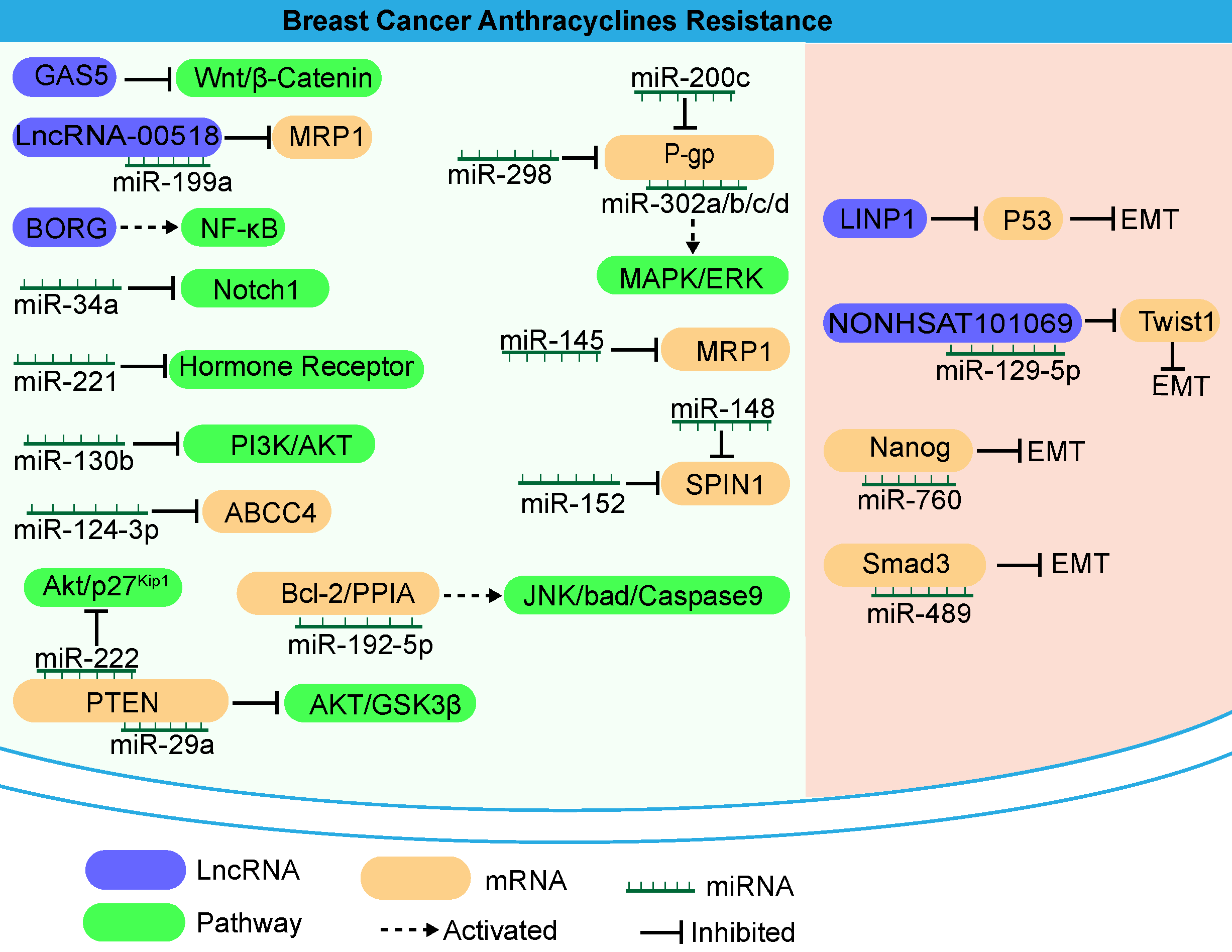

Supplement: Supplementary Figure 1 — The pattern diagram of ncRNAs and Anthracyclines chemoresistance. [file Image_1.tif]

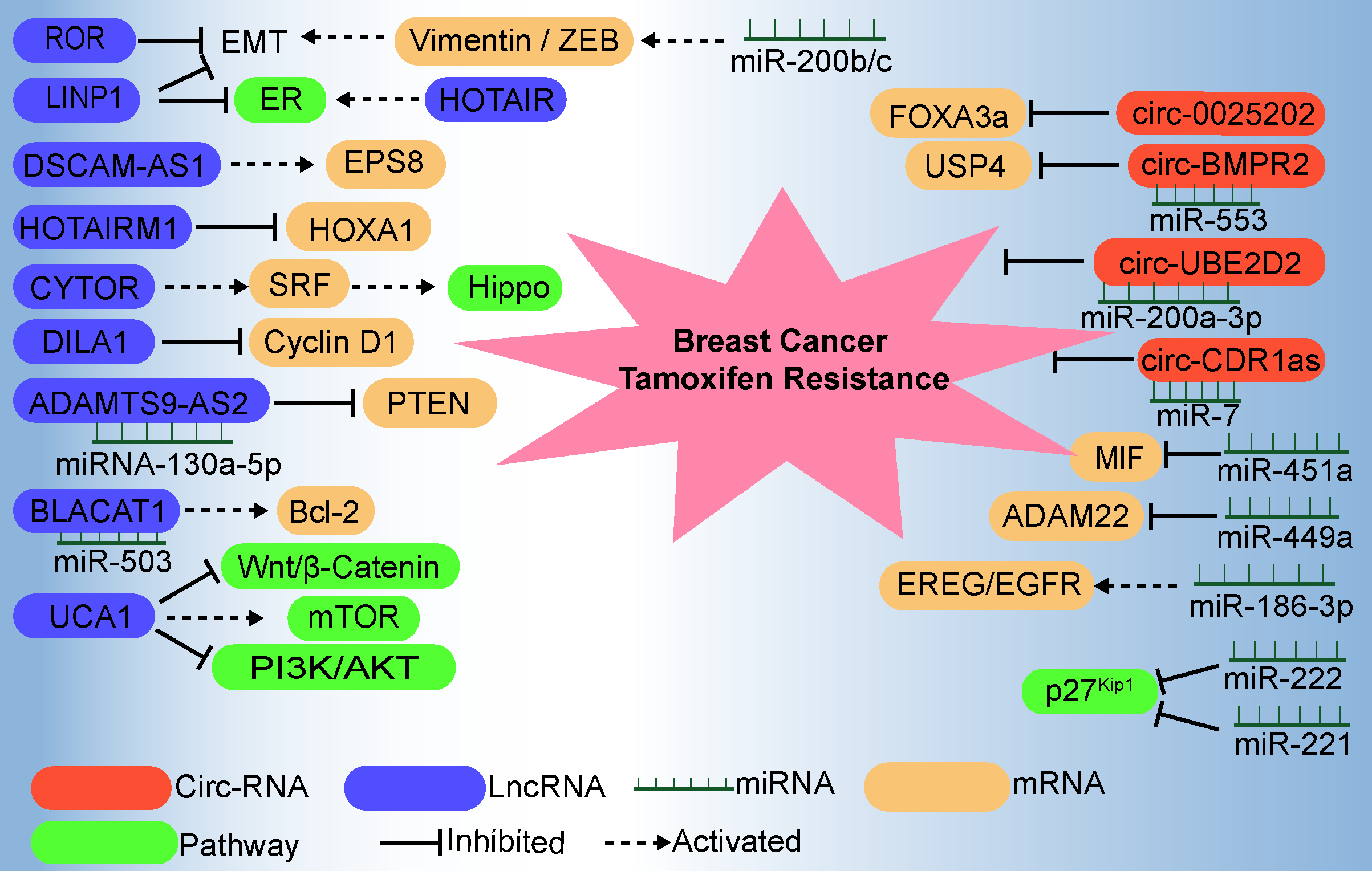

Supplement: Supplementary Figure 2 — The pattern diagram of ncRNAs and Tamoxifen chemoresistance. [file Image_2.tif]

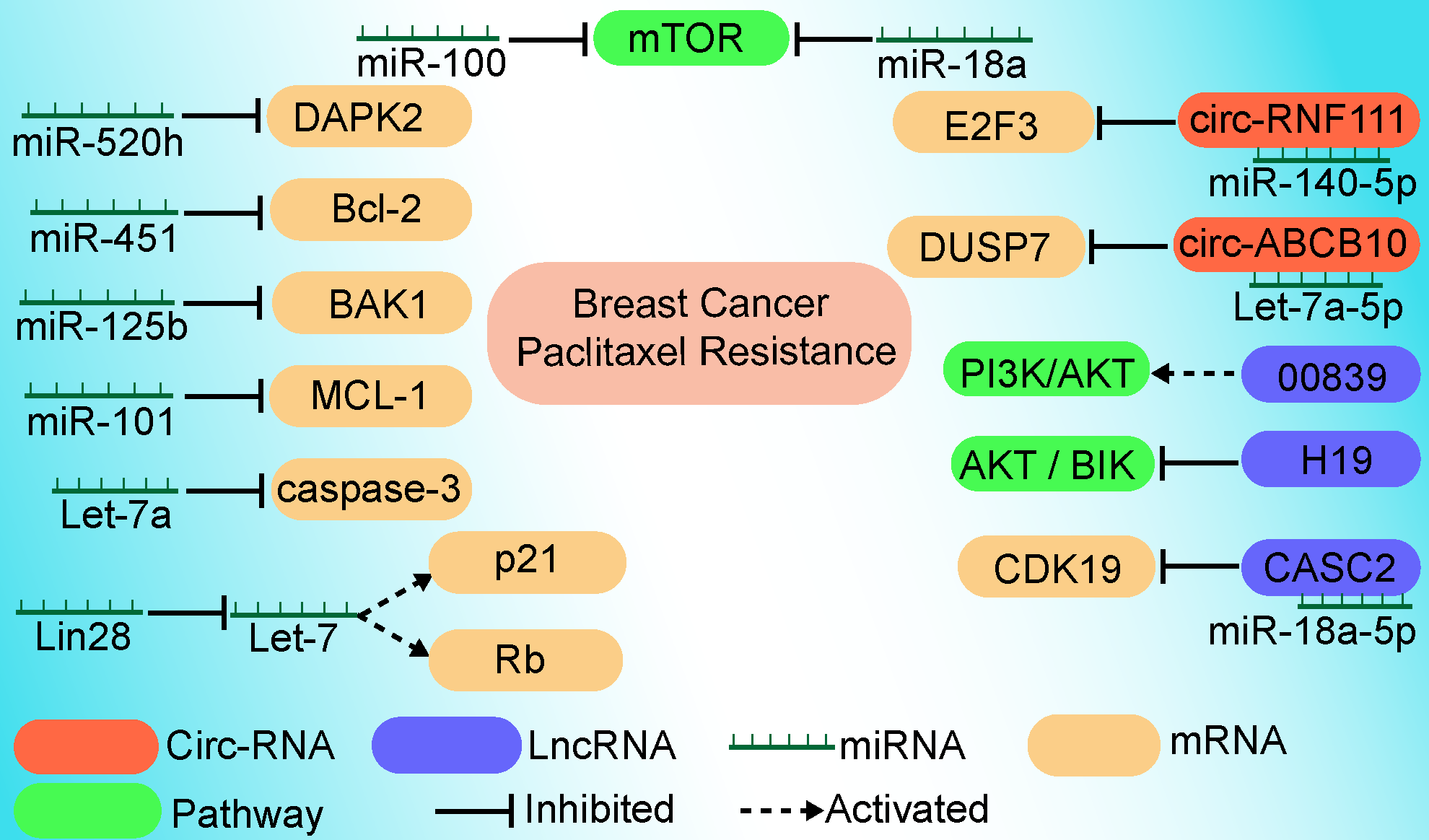

Supplement: Supplementary Figure 3 — The pattern diagram of ncRNAs and Paclitaxel chemoresistance. [file Image_3.tif]

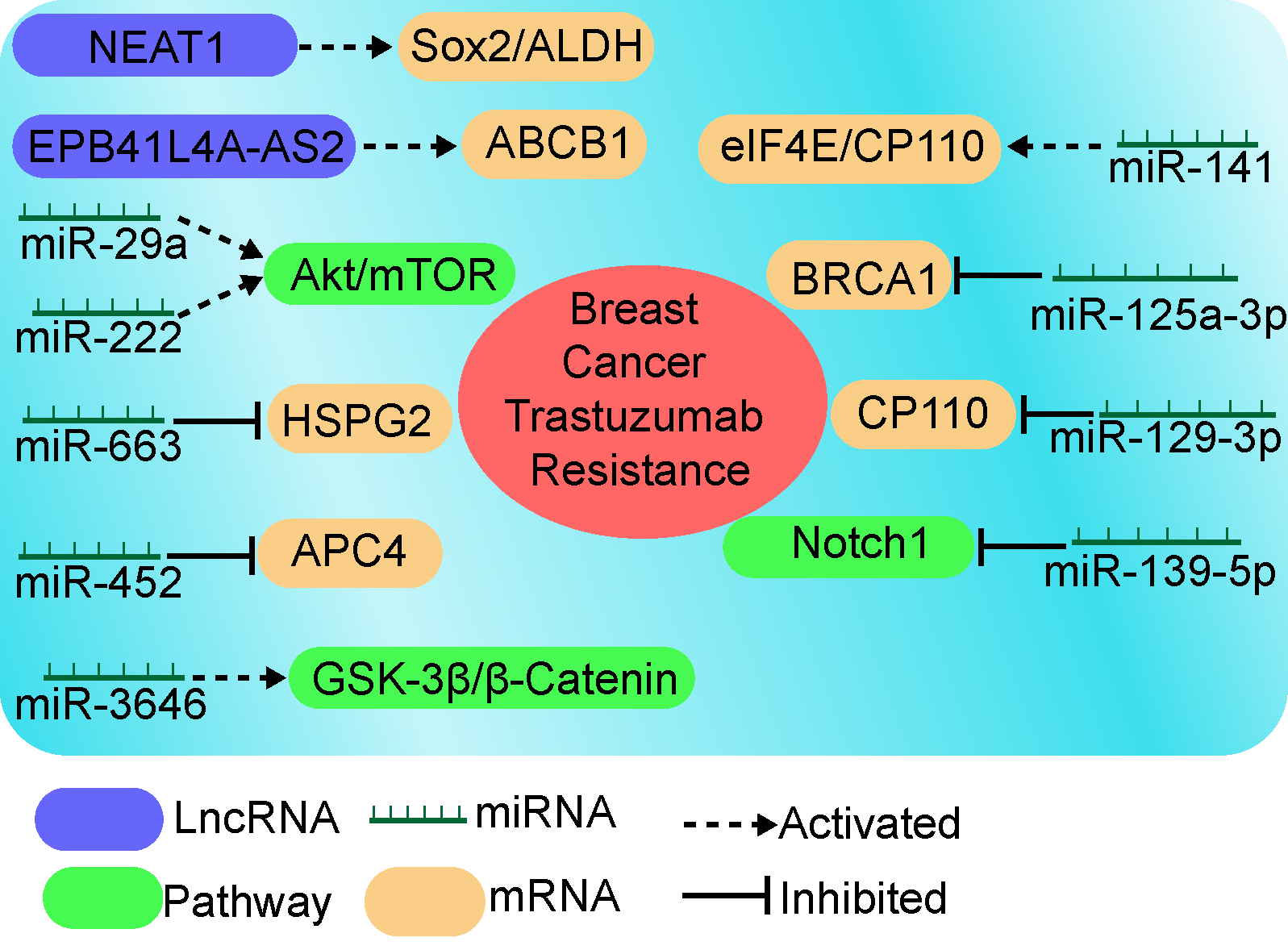

Supplement: Supplementary Figure 4 — The pattern diagram of ncRNAs and Docetaxel chemoresistance. [file Image_4.tif]

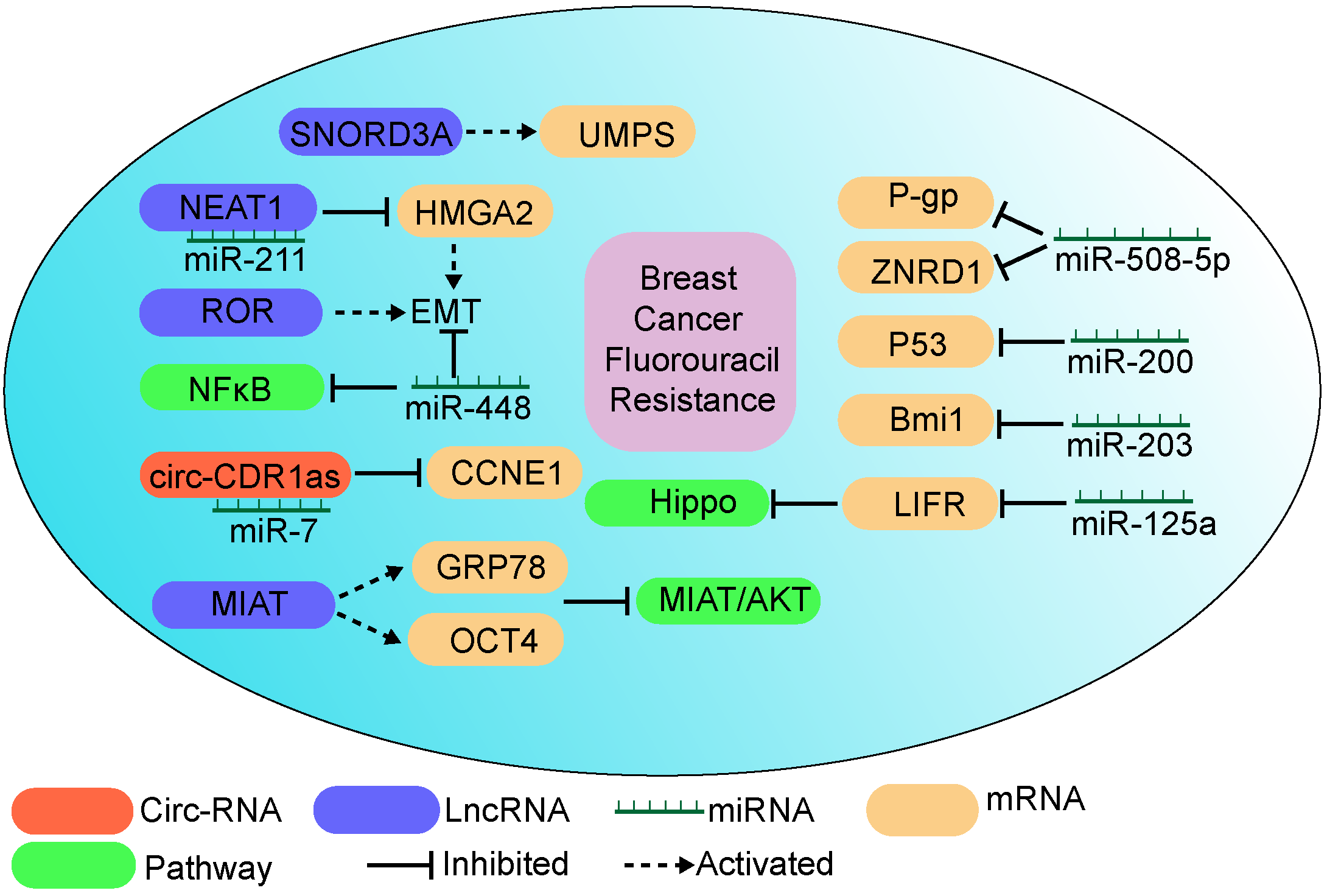

Supplement: Supplementary Figure 5 — The pattern diagram of ncRNAs and Fluorouracil chemoresistance. [file Image_5.tif]

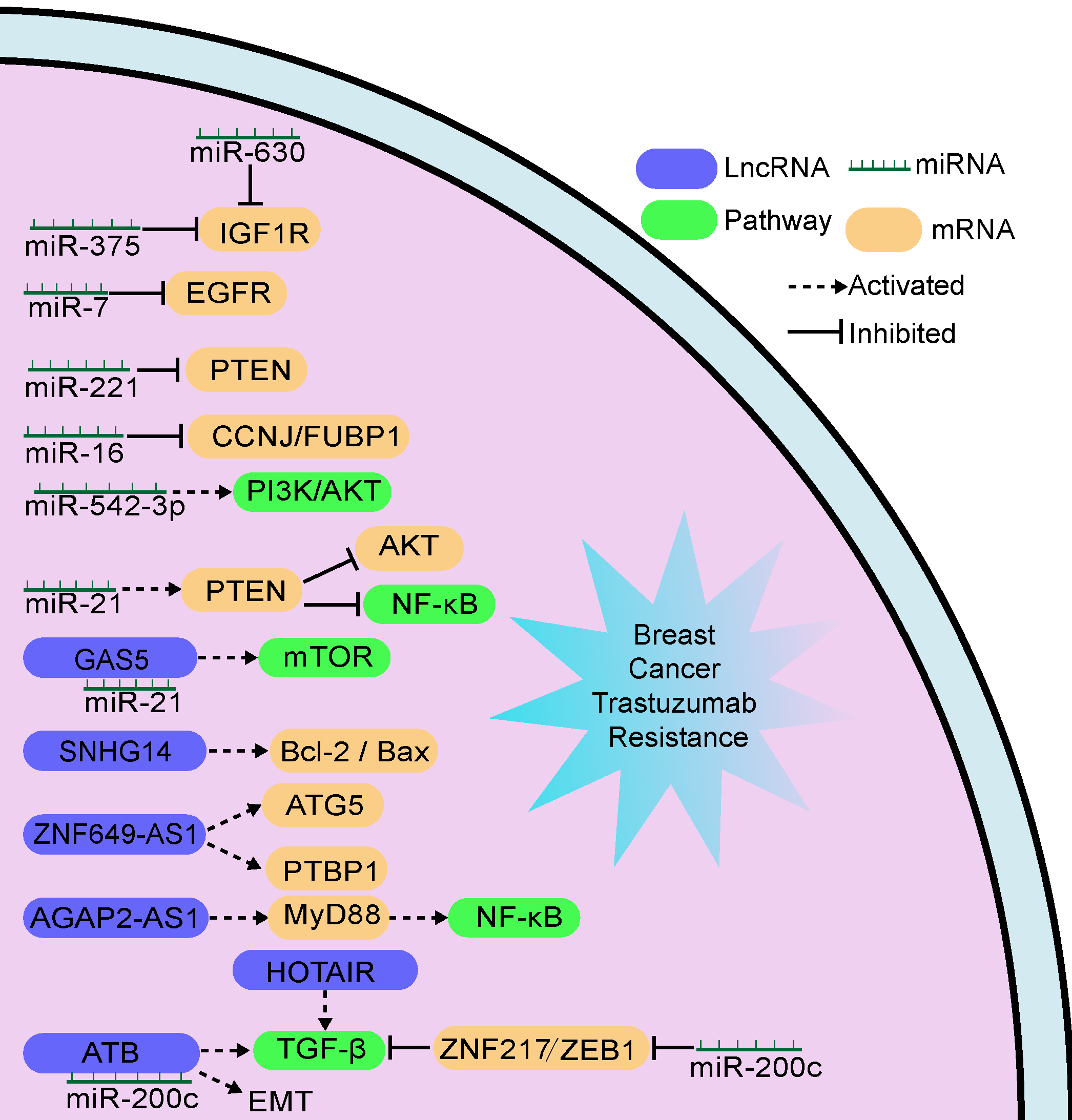

Supplement: Supplementary Figure 6 — The pattern diagram of ncRNAs and Trastuzumab chemoresistance. [file Image_6.tif]
